# The complete chloroplast genome sequence of the *Siraitia Grosvenorii* (Cucurbitaceae)

**DOI:** 10.1080/23802359.2019.1624636

**Published:** 2019-07-11

**Authors:** Qianglong Zhu, Xingyue Liu, Putao Wang, Tianxu Cao, Nan Shan, Qinghong Zhou

**Affiliations:** Department of Horticulture, College of Agronomy, Jiangxi Agricultural University, Nanchang, P.R. China

**Keywords:** *Siraitia grosvenorii*, chloroplast genome, luo-han-guo, monk fruit

## Abstract

*Siraitia grosvenorii* is a famous Chinese plant used in traditional food and medicine with pharmacological effects. The complete chloroplast genome sequence of *S. grosvenorii* has been determined in this study. The total genome size is 158,834 bp in length and contains a pair of inverted repeats (IRs) of 26,288 bp, which were separated by large single-copy (LSC) and small single-copy (SSC) of 87,702 bp and 18,556 bp length, respectively. A total of 131 genes were predicted including 86 protein-coding genes, 37 tRNA genes, and 8 rRNA genes. Phylogenetic analysis showed that *S. grosvenorii* belongs to the family Cucurbitaceae. The complete chloroplast genome of *S. grosvenorii* would play a significant role in the development of molecular markers in plant phylogenetic and population genetic studies.

*Siraitia grosvenorii* (Swingle) C. Jeffrey, commonly known as ‘luo-han-guo’ or ‘monk fruit’, is a famous herbaceous perennial plant in Cucurbitaceae and commonly used for food and medicine materials in China (Xia et al. 2018). Mature fruit of *S. grosvenorii* contains abundant mogrosides and other secondary metabolites, which have great pharmacological effects on lung congestion, sore throat, and constipation (Chun et al. 2014). It is a natural nonsugar sweetener beneficial to human health, which has a sweetening strength of ∼300 times that of sucrose (Zhang et al. 2016). In addition, monk fruit products have been approved as dietary supplements in America, Australia, and Japan (Xia et al. 2018). Chloroplast genome is important for plant photosynthesis and species classification, few genes in the chloroplast genome of *S. grosvenorii* have been applied for analyzing phylogenetic relationship in Cucurbitaceae (Kocyan et al. 2007; Schaefer et al. 2009), but the chloroplast genome of *S. grosvenorii* have not been reported to date. Therefore, we reported the complete sequence of chloroplast genome of *S. grosvenorii* with a hope to promote these researches based on its chloroplast genome in this study.

Sample of *S. grosvenorii* (accession no. JXAU-Sg01) was stored in Jiangxi Agricultural University (28°45′27″N, 115°50′20″E), Nanchang, China. The genomic DNA was isolated from the leaves of *S. grosvenorii* using the CTAB method as previously described (Itkin et al. 2016). Genomic DNA was subjected to construct a ∼470 bp pair-end library and sequenced by Illumina HiSeq 2500 (BioMarker, Beijing, China). About 2 Gb of sequence data were obtained after sequencing and base quality control, clean pair-end reads (2 × 100 bp) of 327 Mb were randomly extracted using Seqtk and assembled with using the Plasmidspades.py in SPAdes (v3.10.1) (Bankevich et al. 2012). Contigs representing the chloroplast genome were retrieved, ordered, and joined into a single draft sequence by comparison with the chloroplast genome of *Cucurbita pepo* (NC_038229.1) as a reference (Zhang et al. 2018). The gaps in the single draft sequence were closed using GapCloser (v1.12-r6). The draft sequence was then confirmed and manually corrected by pair-end read mapping. Finally, the complete sequence was annotated using the two integrated web servers, Geseq (Tillich et al. 2017) and DOGMA (Wyman et al. 2004), and manually checked and corrected by Sequin.

The complete chloroplast genome of *S. grosvenorii* (accession no. MK818498) is 158,834 bp in length with 36.88% GC contents, and exhibits a typical quadripartite structure, consisting of a pair of inverted repeat regions (IRs, 26,288 bp) separated by the large single-copy (LSC, 87,702 bp) and small single-copy (SSC, 18,556 bp) regions. There is a total of 131 genes, including 85 protein-coding genes, 8 rRNA genes, and 37 tRNA genes; six of the protein-coding genes, six of the tRNA genes, and four rRNA genes are duplicated within the IRs.

To determine the phylogenetic position of *S. grosvenorii*, a phylogenetic analysis was conducted with 16 complete chloroplast genomes, 15 of these belonged to Cucurbitaceae and one to *Vitis ninifera* which is considered an outgroup. The phylogenetic tree was constructed by Maximum Likelihood method using MAFFT v7.407 (Nakamura et al. 2018) and MEGA-X (Kumar et al. 2018). The tree showed that *S. grosvenorii* belonged to Cucurbitaceae, and was closer to *Momordica charantia* and *Gynostemma pentagynum*, but has remote phylogenetic relationship with these genera, e.g. *Citrullus* and *Cucumis* (Figure 1). The conclusions further support the previous research results (Schaefer et al. 2009).

**Figure 1.**
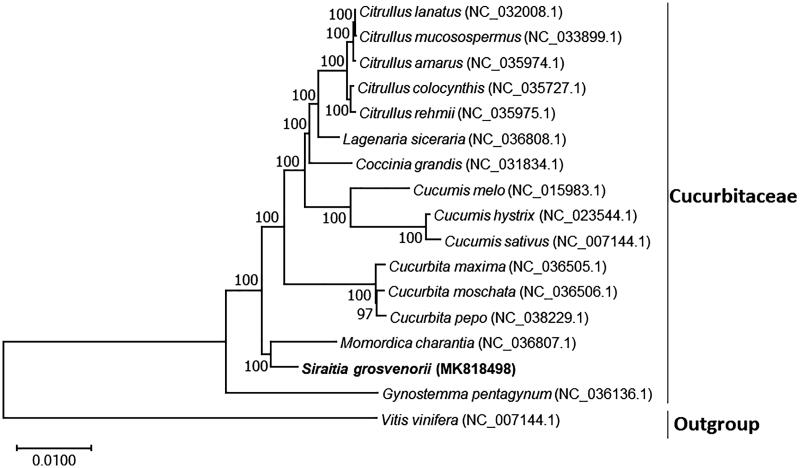
Phylogenetic tree showing relationship between *S. grosvenorii* and 15 species belonging to Cucurbitaceae family, *V. vinifera* was taken as the outgroup. Phylogenetic tree was constructed based on the complete chloroplast genomes using maximum likelihood (ML) with 1000 bootstrap replicates. Numbers in each the node indicated the bootstrap support values.
